# Metastatic Lesions to the Liver

**DOI:** 10.1155/2013/104365

**Published:** 2013-11-17

**Authors:** Jorge Ortiz, David K. Imagawa, Roberto Verzaro, Francesco Serafini, Liise K. Kayler

**Affiliations:** ^1^Einstein Medical Center, USA; ^2^Department of Surgery, University of California, USA; ^3^Surgery Department ISMETT, Italy; ^4^Downstate Medical Center, USA; ^5^Department of Surgery, University of Florida Medical Center, USA

Welcome to this issue of International Journal of Hepatology. The focus will be on metastatic lesions to the liver. We are hopeful that this topic will be of interest to hepatobiliary surgeons, surgical oncologists, medical oncologists, and CT, MR, and interventional radiologists.

Liang et al. review hepatic arterial infusion for liver lesions. This therapeutic modality has been propounded since the 1990s by Margaret and Nancy Kemeny, a surgical and medical oncologist, respectively. Liang et al. relate that hepatic arterial infusion may provide good locoregional control of liver tumors. It may be particularly beneficial for colorectal tumors in combination with systemic chemotherapy. Exciting new medications infused through the device may one day lead to stunning advances in this field.

Kelly et al. review hepatobiliary contrast-enhanced magnetic resonance imaging for liver metastasis from colorectal cancer. They provide a succinct summary of this topic which should improve the surgeon's ability to plan the appropriate surgical approach and consent patients correctly.

Kele et al. revealed a lack of anatomical concordance between preablation and postablation CT images, which may be a risk factor related to ablation site recurrence. This is very important since accurate ablation of metastatic lesions remains the cornerstone of excellent long-term outcomes. Additionally, early detection of recurrence and risk factors for said recurrence are necessary in order to initiate timely retreatment.

Bacchetti et al. review, via a meta-analysis,surgical treatment and survival for metastatic neuroendocrine tumors. They found a significantly longer survival in patients treated surgically compared to those treated with embolization. This meta-analysis is another example of advances made in this field including transplantation and new medications.

Nguyen-Khac et al. review hepatotoxicity (particularly sinusoidal dilatation) associated with chemotherapy. This is particularly important since many patients receive many courses of chemotherapy before and after surgical intervention. Hopefully, with the advent of new medications, these side effects will be attenuated.

We believe this issue is just the beginning in a long line of special issues which will lead to greater understanding of complex hepatologic issues and improved outcomes for our patients.

